# Anterior Mediastinal Benign Teratodermoid Tumour With Intraparenchymal Extension Through Cystobronchial Connection: A Rare Case Report

**DOI:** 10.7759/cureus.23030

**Published:** 2022-03-10

**Authors:** Rupesh Kumar, Vikram Halder, Soumitra Ghosh, Krishna Prasad Gaurav, Debajyoti Chatterjee, Harkant Singh

**Affiliations:** 1 Department of Cardiothoracic Surgery, Postgraduate Institute of Medical Education and Research (PGIMER), Chandigarh, IND; 2 Department of Cardiology, Postgraduate Institute of Medical Education and Research (PGIMER), Chandigarh, IND; 3 Department of Pathology, Postgraduate Institute of Medical Education and Research (PGIMER), Chandigarh, IND

**Keywords:** anterior mediastinal mass, cystobronchial connection, teratodermoid tumour, benign tumour, mature cystic teratoma

## Abstract

Asymptomatic presentation is common in benign mature mediastinal tumours. Symptoms of the above diseases are sometimes life-threatening and can cause massive hemoptysis, recurrent pulmonary infection, hypoxia related to the pulmonary parenchymal hemorrhage, or pressure effect on or more of the major bronchi. A 16-year-old boy presented with frequent episodes of hemoptysis and recurrent fever unresponsive to antimicrobials. On investigation, it was found to be a benign mature mediastinal mass with cystobronchial connection to the right middle lobe. Resection of this mass resulted in the complete recovery of the individual. An anterior mediastinal benign teratodermoid tumour with intraparenchymal extension through cystobronchial connection is very rare. Surgical resection is challenging but offers the complete cure.

## Introduction

Only 15% of the anterior mediastinal mass is a germ cell tumour. The typical presenting age is the second and fourth decade. Nonseminomatous tumour is more common than the seminomatous tumour in the anterior mediastinum [1]. These tumours are commonly asymptomatic, and compression symptoms are rare due to protrusion (left side > right side). Rare presentation of mediastinal germ cell tumour includes compression, rupture into lung parenchyma, and fistula to bronchus and skin [2,3]. We are presenting a case of mediastinal mass (mature cystic teratoma) with intraparenchymal extension through cystobronchial connection with right middle lobe bronchus, managed with mediastinal mass excision with right middle lobectomy.

## Case presentation

A 16-year-old non-smoker male presented to the ED with cough and frequent episodes of hemoptysis. On clinical examination, the patient had a respiratory rate of 18/min, oxygen saturation (SpO2) of 98% in room air, and normal bilateral vesicular breath sound with no added sound. After initial resuscitation, hemoptysis was controlled.

**Figure 1:**
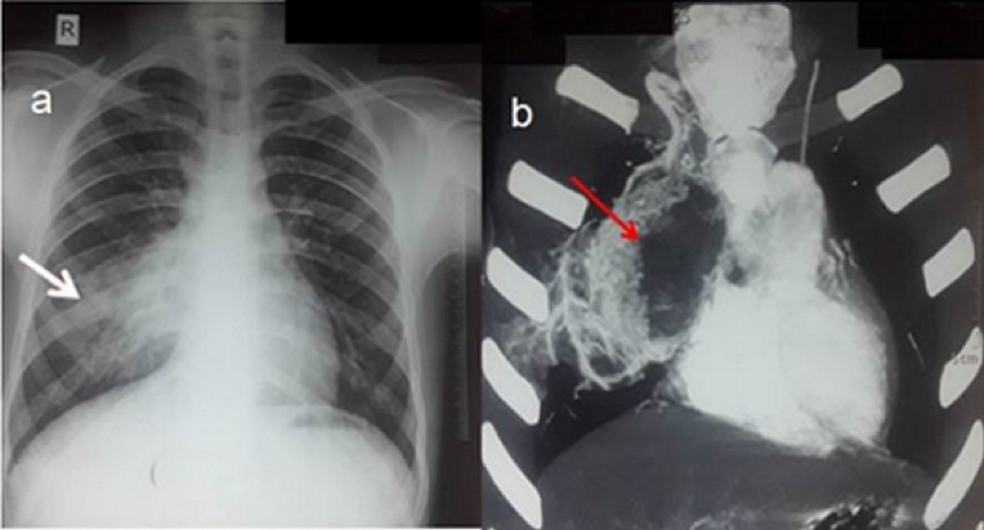
(a) Chest X-ray showing an ill-defined mass in the right hilar region. (arrow showing perihilar mass). (b) Contrast-enhanced CT of thorax showing an 8x8 cm heterogeneous mass in anterior mediastinum compressing the right atrium and abutting the right ventricle (arrow showing mediastinal mass).

On chest X-ray (CXR), ill-defined opacity in the right hilar and para hilar region was seen (Figure 1a). CT scan revealed an 8x8 cm heterogeneous mass in the anterior mediastinum without any lymphadenopathy. CT angiography (CTA) revealed that mass to be connected to the right bronchial tree but no connection with any blood vessel (Figure 1b). On blood investigation, alpha-fetoprotein (AFP), lactate dehydrogenase (LDH), and beta-human chorionic gonadotropin (β-hCG) were normal. On fiber-optic bronchoscopy, there was evidence of hemoptysis, and communication between the right bronchus intermedius and the cyst was seen.

**Figure 2:**
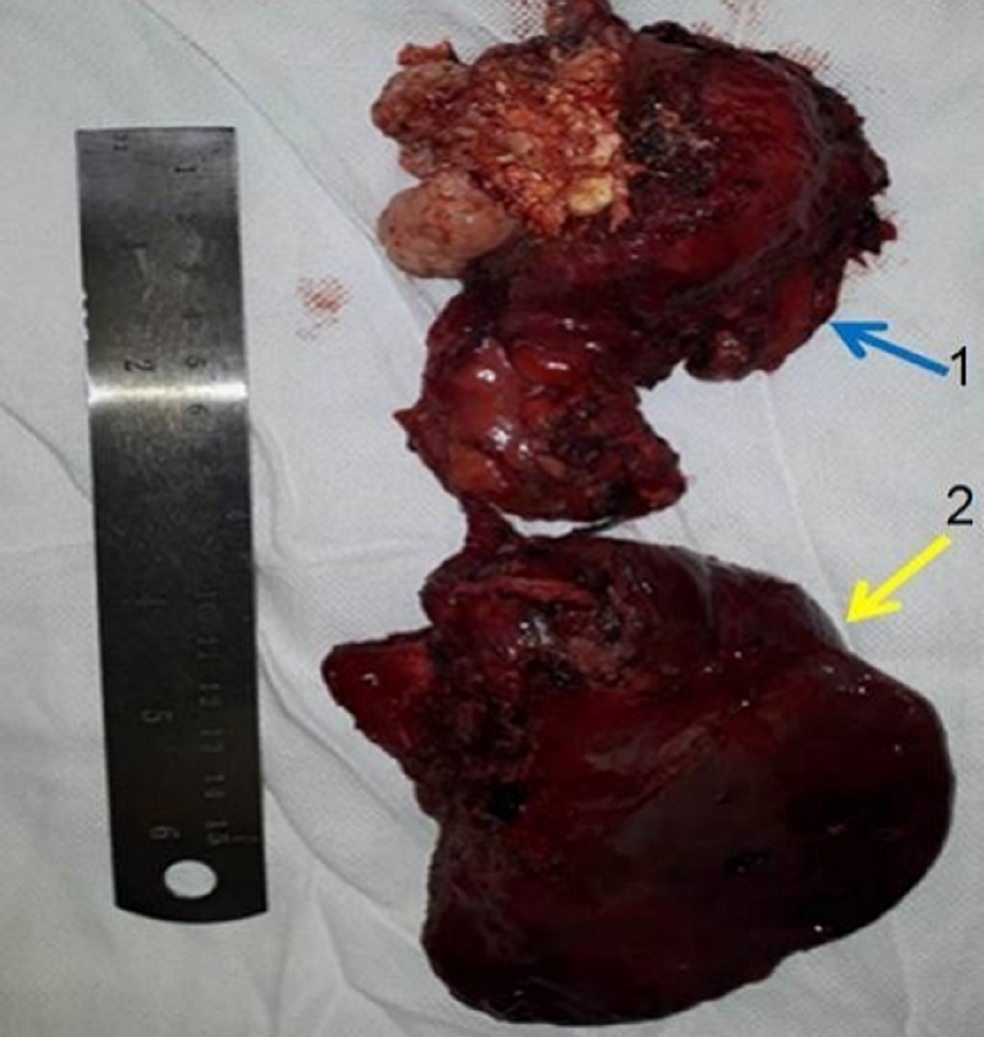
Specimen showing anterior mediastinal mass with cystobronchial communication to the right middle lobe (1: anterior mediastinal mass, 2: middle lobe of the right lung).

The patient was taken for surgery, and after sternotomy, mass was dissected from the adjacent right atrium and aorta. Mass was resected, cystobronchial communication was identified, and right middle lobectomy was done after dissection and ligation of hilar structures (Figure 2).

Post-surgery, the patient was extubated after six hours. In post-operative CXR, there was no pleural or mediastinal collection or lung atelectasis. Intercostal drain (ICD) was removed after three days. The total duration of hospital days was seven days, including three days in the critical care unit.

**Figure 3:**
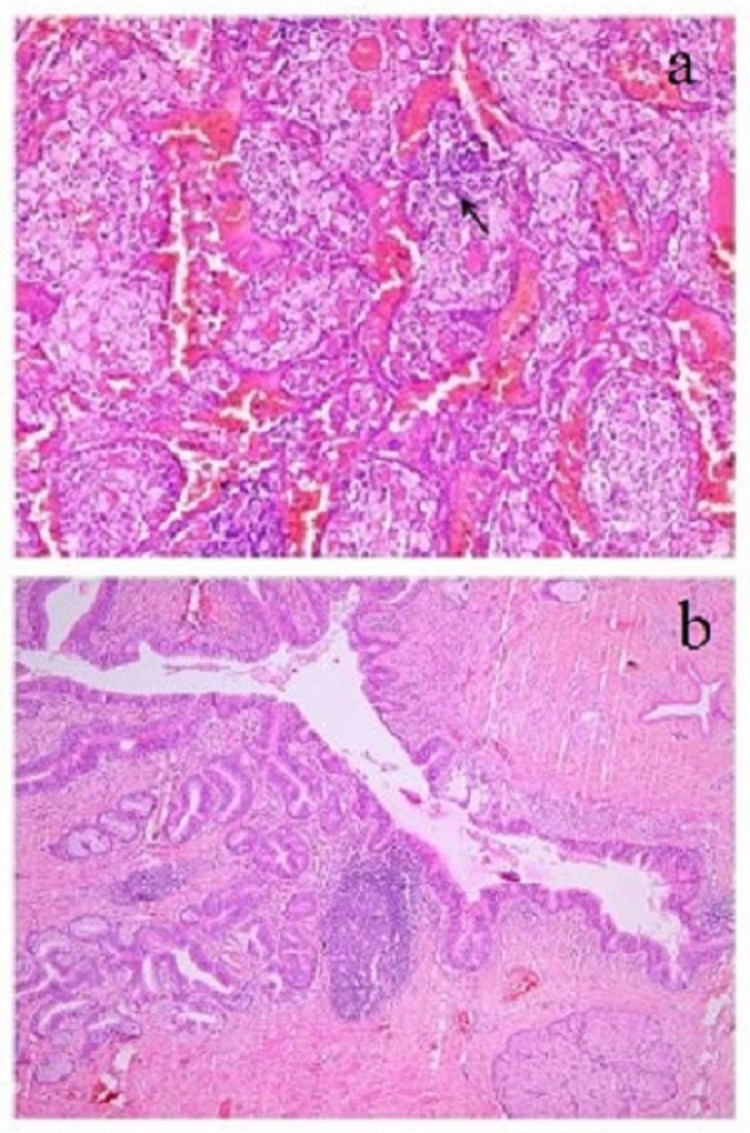
(a) A histological examination from the mediastinal mass shows a cyst lined by the respiratory and intestinal epithelium. The stroma shows the presence of smooth muscle, sebaceous gland, lymphoid aggregates, and fibrous tissue (H&E, x40) (b) Section examined from lung shows expanded alveolar spaces filled with foamy macrophages, indicating lipoid pneumonia due to obstruction (H&E, x100).

A histological examination of the mediastinal mass shows a cyst lined by the respiratory and intestinal epithelium. The stroma shows the presence of smooth muscle, sebaceous gland, lymphoid aggregates, and fibrous tissue. Section examined from the lung shows expanded alveolar spaces filled with foamy macrophages, indicating lipoid pneumonia due to obstruction (Figure 3). 

**Figure 4:**
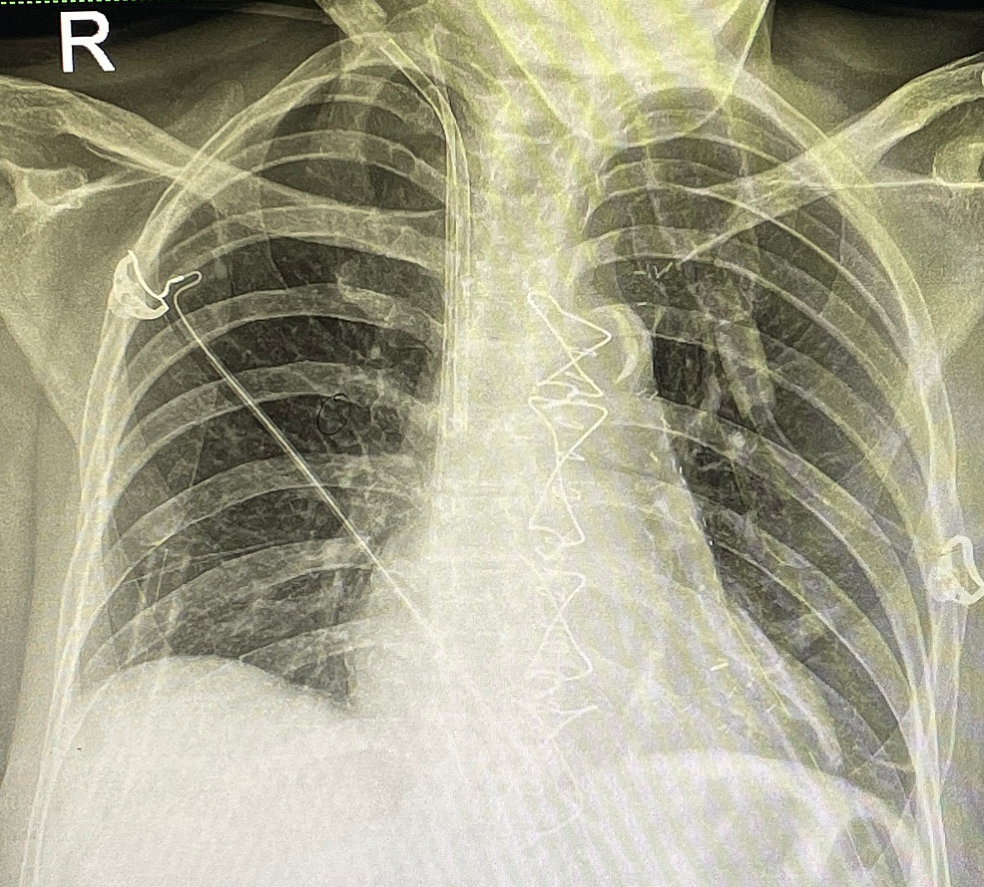
Postoperative chest X-ray showing no recurrent lesion.

The patient was asymptomatic during a three-month follow-up and without any recurrent lesion on CXR (Figure 4).

## Discussion

The most common extragonadal site of germ cell tumour is the anterior mediastinum [2]. The most common mediastinal germ cell tumour is teratoma without any significant gender predilection. Some potential stem cells are shed during thymus primordia development in the embryonic stage. These cells undergo spontaneous vascular development. Germ cell tumours are derived from these cells. It usually contains tissues derived from the ectoderm, mesoderm, and endoderm. Tissues derived from at least two germ layers are needed for pathological diagnosis. According to the degree of differentiation, it is divided into mature teratoma and immature teratoma [3]. Tumour necrosis and superinfection commonly lead to fistula formation [4].

As the most common presentation is asymptomatic, most cases are diagnosed during CXR and CT scans. Chest tightness, dyspnea, neck mass, superior mediastinal syndrome, and Horner syndrome may be presenting symptoms due to compression. The patient may present with pleural effusion and hemothorax, which cause dyspnea, hemoptysis, obstructive pneumonia, and pericardial effusion and tamponade. On CXR and CT scan, partial lobulated and calcified round mass is present in the anterior mediastinum. Tooth and bone can be found in the tumour. Mature teratomas are mostly cystic masses, while immature teratomas are primarily solid masses. CT is the primary imaging modality to diagnose bronchial tree fistulas and delineate possible causes like an abscess, pneumonia, and tumour. In the case of hemoptysis, CTA is useful to identify vessels, and in case of emergency, embolization can be done. AFP, β-HCG, and LDH are used to diagnose and monitor cases [5].

Surgical resection is an effective treatment in benign mature teratoma. However, due to extensive adhesion, large wound surface, large size, incomplete lung expansion, and residual cavity in the chest cavity, dissection is challenging, and postoperative bleeding chances are higher. During dissection, adequate care should be given to protect the phrenic nerve, recurrent laryngeal nerve, vagus nerve, and brachial plexus nerve in case of large adherent mass. If the middle mediastinum, innominate vein, superior vena cava, head and neck vessels are involved, it is advisable to choose median sternotomy for surgical resection when vascular reconstruction is needed. When the tumour extends to neck through the thoracic outlet, a “T” shaped incision should be made through the median sternum combined with the neck collar incision. A posterolateral thoracotomy may be preferred when wedge-shaped resection of the lung, lobectomy, or pneumonectomy is needed. For large tumour, lung, and pericardial invasion due to the perforation of the surrounding tissues or dense adhesion, partial pericardiectomy, pulmonary lobectomy, and wedge-shaped excision of the lung are done [6]. Thoracoscopic resection of mediastinal teratoma can be done if the maximum diameter of tumour is less than 10 cm, without estimated adhesions from preoperative imaging to reduce intraoperative blood loss and shorten postoperative hospital stay [7].

## Conclusions

Anterior mediastinal benign teratodermoid tumour with intraparenchymal extension through cystobronchial connection is very rare. CXR and CT thorax are used to diagnose it. Surgical resection is complicated, but it offers the complete cure. Blood LDH, β-hCG, AFP are used to monitor the response to therapy. Histopathological diagnosis is confirmatory.
